# Interactive effects between extreme temperatures and PM_2.5_ on cause-specific mortality in thirteen U.S. states

**DOI:** 10.1088/1748-9326/ad97d1

**Published:** 2024-12-06

**Authors:** Edgar Castro, James Healy, Abbie Liu, Yaguang Wei, Anna Kosheleva, Joel Schwartz

**Affiliations:** Harvard T.H. Chan School of Public Health, Boston, MA, United States of America

**Keywords:** temperatures, PM2.5, extreme heat, extreme cold, mortality, interaction

## Abstract

The extent and robustness of the interaction between exposures to heat and ambient PM_2.5_ is unclear and little is known of the interaction between exposures to cold and ambient PM_2.5_. Clarifying these interactions, if any, is crucial due to the omnipresence of PM_2.5_ in the atmosphere and increasing scope and frequency of extreme temperature events. To investigate both of these interactions, we merged 6 073 575 individual-level mortality records from thirteen states spanning seventeen years with 1 km daily PM_2.5_ predictions from sophisticated prediction model and 1 km meteorology from Daymet V4. A time-stratified, bidirectional case-crossover design was used to control for confounding by individual-level, long-term and cyclic weekly characteristics. We fitted conditional logistic regressions with an interaction term between PM_2.5_ and extreme temperature events to investigate the potential interactive effects on mortality. Ambient PM_2.5_ exposure has the greatest effect on mortality by all internal causes in the 2 d moving average exposure window. Additionally, we found consistently synergistic interactions between a 10 *μ*g m^−3^ increase in the 2 d moving average of PM_2.5_ and extreme heat with interaction odds ratios of 1.013 (95% CI: 1.000, 1.026), 1.024 (95% CI: 1.002, 1.046), and 1.033 (95% CI: 0.991, 1.077) for deaths by all internal causes, circulatory causes, and respiratory causes, respectively, which represent 75%, 156%, and 214% increases in the coefficient estimates for PM_2.5_ on those days. We also found evidence of interactions on the additive scale with corresponding relative excess risks due to interaction (RERIs) of 0.013 (95% CI: 0.003, 0.021), 0.020 (95% CI: 0.008, 0.031), and 0.017 (95% CI: −0.015, 0.036). Interactions with other PM_2.5_ exposure windows were more pronounced. For extreme cold, our results were suggestive of an antagonistic relationship. These results suggest that ambient PM_2.5_ interacts synergistically with exposure to extreme heat, yielding greater risks for mortality than only either exposure alone.

## 1. Introduction

Adverse temperatures and fine particulate matter (PM_2.5_) are both universally experienced, widely understood to be harmful to human health, and associated with significant mortality burdens globally (Gasparrini *et al* 2015, Liu *et al* 2019, Zhao *et al* 2021). In 2019, 6.45 million deaths (82.8 per 100 000) were attributed to PM_2.5_ exposure, while 1.96 million deaths (25.7 per 100 000) were attributed to exposure to adverse temperatures (Murray *et al* 2020). Accordingly, over the past few decades, both exposures have received increased attention from both the scientific community and the general public, especially as extreme heat events have become more common (Collins *et al* 2000, Donat *et al* 2013, Kruger and Sekele 2013). Much attention has been given also to global warming as changes in the climate become more apparent and as the rate of warming increases (IPCC 2022).

Though the separate effects of adverse temperatures and PM_2.5_ have been well established, less is known of the synergism between the two. Such information is particularly relevant for issues related to health disparities and environmental justice, as disadvantaged communities are often exposed to both (Eanes *et al* 2020, Benz and Burney 2021, Jbaily *et al* 2022, Renteria *et al* 2022), for example neighborhoods that have been redlined (Lane *et al* 2022, Li *et al* 2022, Wilson 2020). Some past work has been done to investigate modification of the effects of PM_2.5_ by ambient temperature, finding that exposures to both non-optimal temperatures and more temperature variability increase the risk of PM_2.5_ on adverse outcomes such as mortality and hospitalization (Kioumourtzoglou *et al* 2015, Li *et al* 2015, Liu *et al* 2019, Steenland *et al* 2022). Other studies focused specifically on the interactive effects of exposures to both adverse temperature conditions and ambient PM_2.5_, finding significant positive synergism between the two exposures (Huang *et al* 2023, Ji *et al* 2020, Xu *et al* 2023, Yitshak-Sade *et al* 2018, Zhou *et al* 2023). However, many studies examined exposures at the city level, which ignores important within-city variations in both exposures. This approach also ignores deaths occurring in smaller cities, towns, and rural areas. Further, few studies have looked specifically at the interactions between PM_2.5_ and local temperature extremes, which are more severe, vary by location, and may behave differently than adverse temperatures closer to the mean daily temperatures, especially those extreme conditions that last for multiple days (Hajat *et al* 2006).

One study in China avoided these issues by using gridded PM_2.5_ and metrological predictions at 1 km and 0.0625° resolutions, respectively, additionally using local, grid cell-based definitions of ‘extreme’ and a comprehensive record of mortality from Jiangsu Province (Xu *et al* 2023). That study found significant interactions between PM_2.5_ and both extreme heat and cold events on risk of mortality by myocardial infarction. However, few similar studies have been done in the United States. A recent study from California also used systematic death records and spatially local extreme heat definitions, finding that risks of mortality were increased by co-exposures to extreme ambient PM_2.5_ concentrations and extreme heat (Azzouz *et al* 2024), but that study dichotomized PM_2.5_ concentrations, precluding per-unit estimates of risk and was additionally limited only to California.

Many of these past studies also did not consider the effects of adaptation, wherein the effect of a given adverse temperature on a given population becomes attenuated over time due to adaptive changes in that population to its local climate (Davis *et al* 2002). Some examples of adaptation include physiological acclimatization, changes in behavior, and installation of adverse temperature-mitigating equipment such as air conditioners and heaters. As a result, reported estimates of the interaction between extreme heat and PM_2.5_, for example, may be over-reported in earlier years and under-reported in recent years owing to rising global temperatures due to climate change, or the effect may also have changed over time. One recent study in California investigated exposures to both extreme temperatures and extreme ambient PM_2.5_ levels by dichotomizing both exposures, finding that exposure to both increased mortality beyond exposure to either individually (Rahman *et al* 2022). Although this study did consider spatial and temporal adaptation, it did not consider extreme cold temperatures, and the dichotomization of PM_2.5_ precluded estimates of increased risk per unit increase in PM_2.5_.

In this study, we aimed to clarify the robustness and extent of the synergistic interaction between PM_2.5_ and adverse ambient temperature exposures, focusing specifically on exposures to ambient temperature extremes. We investigated this interaction using 9.6 million geocoded mortality records from thirteen U.S. states across 16 years and exposure data from high-resolution PM_2.5_ and meteorological models while accounting for spatial variation in exposure, also including small cities, towns, and rural areas, and accounting for adaptation to both differing localized climates and the warming of the planet over time in the process.

## 2. Methods

### 2.1. Data sources

We obtained individual-level mortality records that included information on age, sex, race, education, and residential geographic information from the state departments of public health of California, Florida, Georgia, Illinois, Indiana, Kansas, Massachusetts, Michigan, Missouri, New Hampshire, New Jersey, Ohio, and Texas (table 1). Findings and conclusions are those of the authors and do not necessarily represent the positions of those departments. We restricted our study to records involving individuals 18 yrs or older at the time of death to isolate adult mortality cases and categorized deaths as being due to all internal causes (ICD10 codes A00-R99), circulatory diseases (ICD10 codes I00-I99), respiratory diseases (ICD10 codes J00-J99), or malignant neoplasms (ICD10 codes C00-C97).

**Table 1.** Sources of mortality records.

State	Agency
California	Health and Human Services Agency, Department of Public Health
Florida	Department of Health
Georgia	Department of Community Health, Division of Public Health
Illinois	Department of Public Health, Division of Vital Records
Indiana	Department of Health
Kansas	Department of Health and Environment, Bureau of Public Health Informatics
Massachusetts	Department of Public Health
	Department of Community Health, Division for Vital Records and Health Statistics
Missouri	Department of Health and Senior Services
New Hampshire	Department of Health and Human Services, Division of Public Health Services, Bureau of Public Health Statistics and Informatics
New Jersey	Department of Health and Senior Services, Center for Health Statistics
Ohio	Department of Health
Texas	Department of State Health Services, Center for Health Statistics, Health Information Resources Branch

Records included either the individual’s home address or Census GEOIDs of varying granularity corresponding to the encompassing TIGER/Line geography. We standardized these home locations to Census block groups. For records with block-level GEOIDs (Michigan and New Jersey), we truncated the GEOIDs at the 12th character to obtain Census block group GEOIDs (U.S. Census Bureau 2021). Records with block group-level GEOIDs (Georgia and Michigan) were left as-is. For states that provided home addresses (all other states), we geocoded these addresses to coordinates using ArcGIS Pro and then spatially joined these coordinates to the encompassing Census block group. All records with geographic information coarser than block group-level were left as-is and later excluded.

PM_2.5_ exposures were assessed using daily predictions on a 1 km point grid and at PM_2.5_ monitoring locations from a model that incorporated information from air quality monitors, remotely-sensed satellite data, outputs from two chemical transport models, meteorological data, and land-use data into a geographically-weighted ensemble of machine learners (Di *et al* 2019). This model has previously been shown to have good accuracy with an average cross-validated *R*^2^ of 0.86. Daily PM_2.5_ exposures produced by this model were aggregated to Census block groups also using a two-stage approach. For Census block groups that encompassed one or more centroids of cells from the model’s prediction grid, we took the average of predicted PM_2.5_ values encompassed by that Census block group. For smaller block groups that did not contain any grid cell centroids, we matched the centroids of those block groups to the nearest grid cell centroid by smallest Euclidean distance.

Daily, continuous surface temperature was extracted from the 1 km raster grid outputs of the Daymet V4 surface weather model (Thornton *et al* 2020). We used the *exactextract* program to aggregate vapor pressure and minimum and maximum daily temperatures from Daymet to Census block groups by calculating the area-weighted mean values of grid cells encompassed by each Census block group, weighted by the areas of intersections between block groups and grid cells (Baston 2022, Castro *et al* 2021).

There is no universally agreed-upon cutoff for what constitutes an extreme temperature event (Xu *et al* 2016). For our analyses, we used spatiotemporally-local definitions of extreme: for each block group, we considered days to be ‘extreme heat’ if the minimum temperature was higher than the 99th percentile of minimum temperature in that year in that block group, and days were marked as ‘extreme cold’ if the 5 d moving average of maximum temperature was lower than the 1st percentile of the 5 d moving average of maximum temperature (Schwartz 2005). These time windows were selected based on the existing literature which suggests that the same-day exposure is most relevant for heat-related deaths and that longer moving averages are more relevant for cold-related deaths (Anderson and Bell 2009, Sheridan *et al* 2019).

**Figure 1.**
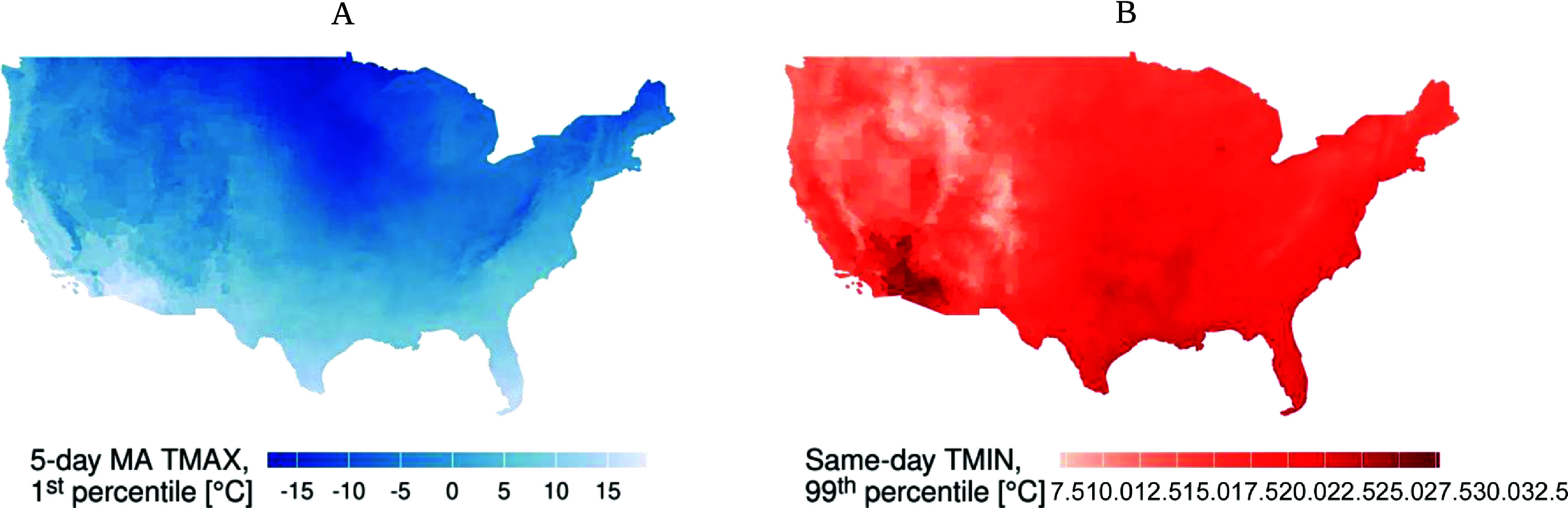
Maps showing (A) the maximum cutoff for the 5 d moving average of maximum temperature (5d MA TMAX) and (B) the minimum cutoff for same-day minimum temperature (same-day TMIN) for a day in the year 2010 to be considered an extreme cold or extreme heat event, respectively, by block group.

These criteria guarantee that the temperature over the course of a given exposure window, which can vary considerably, is at least as extreme as the cutoff value, i.e. that there is no relief. This is in contrast to definitions that utilize the mean or most extreme value, which could be more susceptible to outlying values. These year-by-year, block group-by-block group cutoffs additionally broadly account for area-level trends in adaptation both to differing local climates and to increasing temperatures over time (figure 1), though they do not directly account for specific onsets of individual-level adaptations such as air conditioner use, behavioral changes, changes in clothing, or other individual-level changes which may vary in uptake rate across an area.

In addition to our main exposures, we additionally retrieved tract-level rural-urban commuting area (RUCA) codes from the USDA Economic Research Service for the purposes of assessing effect heterogeneity by rural/urban location (USDA ERS 2023). We truncated each record’s assigned block group-level GEOID from 12 characters to 11 characters to obtain the tract-level GEOID and merged RUCA codes on those, using the year 2000 RUCA Codes for years 2000–2009 and the year 2010 RUCA codes for years 2010–2016. We categorized these RUCA codes into the following groups: metropolitan (primary RUCA codes 1–3), micropolitan (primary RUCA codes 4–6), small town (primary RUCA codes 7–9), and rural (primary RUCA code 10).

We also retrieved Köppen–Geiger climate classifications from the GloH2O project for the purposes of assessing effect heterogeneity by climate zone (Beck *et al* 2023). We assigned Köppen–Geiger classes to Census blocks by matching the centroid of each block to the centroid of the nearest 1 km grid cell from the GloH2O product by Euclidean distance. We then merged population data from the most recent Decennial Census (year 2000 for 2000–2009 and 2010 for 2010–2016), truncated each Census block’s 15-character GEOID to 12-character, block group-level GEOIDs, and determined block group-level Köppen–Geiger classes by choosing which class encompassed the largest proportion of block-level population within each block group. Where there were ties, we instead determined the block group-level Köppen–Geiger class by the largest proportion of block-level land area encompassed. Due to very few records being observed across certain classes, we used only the coarsest classifications (A: Tropical; B: Arid; C: Temperate; D: Cold; and E: Polar).

### 2.2. Statistical analyses

We used a case-crossover design with time-stratified, bidirectional control sampling to control for confounding by fixed and slow-varying individual-level characteristics, cyclic weekly patterns, and longer-term time-varying trends by design (Navidi 1998, Bateson and Schwartz 1999, Sunyer *et al* 2000, Jaakkola 2003). For each case, we created controls by selecting days from the 3 or 4 other days in the same month of the case that were also on the same day of week as the case. By matching on day of the week, were are able to control for cyclic weekly patterns, and by matching on month, we were able to control for seasonal and longer-term trends that do not vary on the scale of a month. By matching on both, we additionally controlled for their interaction, that is, that day of the week effects vary by month. Additionally, because each case also served as their own control, we were able to adjust for all factors that do not vary within an individual on the time scale of a month, such as age, sex, race, pack-years of cigarettes smoked, usual diet, etc, and also stable area-level characteristics. Once controls were created, exposures were assessed separately for each case and control by merging exposure data on each record’s Census block group GEOID and calendar date.

We fit conditional logistic regressions within strata of each individual as follows:

\begin{align*}clogit\left( {Pr\left( {{\text{death}}} \right)} \right) &amp; = {\beta _0} + {\beta _1}{\text{P}}{{\text{M}}_{2.5}} + {\beta _2}{\text{extreme}}\nonumber\\ &amp; \quad + \mathop \sum \limits_{i = 1}^4 {\beta _{2 + i}}n{s_i}\left( {{\text{VP}},df = 4} \right) \nonumber\\ &amp; \quad+ {\beta _7}{\text{P}}{{\text{M}}_{2.5}} \times {\text{extreme}}\end{align*}

Where *extreme* refers to extreme heat or extreme cold; *VP* is vapor pressure; *β*_2*+i*_ are the coefficients for the basis functions of the natural spline of vapor pressure, where each basis function is indexed by *i*; and *β*_7_ is the interaction of interest. Separate models were fit for extreme heat and extreme cold and for each category of deaths. In our main analysis, we focused on the 2 d moving average of PM_2.5_ and the strictest definitions of extreme temperature, i.e. greater than the 99th percentile of minimum temperature for extreme heat and less than the 1st percentile of maximum temperature for extreme cold, respectively. Additionally, since odds ratios from case-crossover designs with informative sampling schemes approximate risk ratios from cohort studies (Navidi and Weinhandl 2002), we computed the relative excess risk due to interaction (RERI) for each model to assess interaction on the additive scale. Confidence intervals for these RERIs were computed using a non-parametric bootstrap approach with 600 bootstrap samples for each estimate. As sensitivity analyses, we considered alternate definitions of extreme heat and cold events, using cutoffs at the 90th and 10th and 85th and 15th percentiles of minimum and maximum temperature for each block group for each year, respectively, and also considered same-day and 3–5 d moving averages of PM_2.5_ (i.e. moving averages up to lag day 4).

We also considered potential effect modification of the primary exposures of interest by sex, race (Black vs. White, as a crude proxy for the experience of racism), age (less than vs. greater than or equal to 65 years old), highest level of educational attainment (less than high school, high school, and greater than high school), urbanicity (by RUCA code group), local annual PM_2.5_ levels (less than vs. greater than or equal to 9 *µ*g m^−3^), state, and Köppen–Geiger classification via stratified analyses. To test whether the main effects and interactions obtained from these analyses were significantly different from each other, we fit random effects meta-regression on the estimated coefficients and corresponding standard errors for each grouping variable. For these tests of heterogeneity, we adjusted our *p*-values using the FDR correction to account for multiple testing of the hypotheses of effect heterogeneity of the two main effects and the interaction by the aforementioned eight grouping variables on the three outcomes studied.

**Figure 2.**
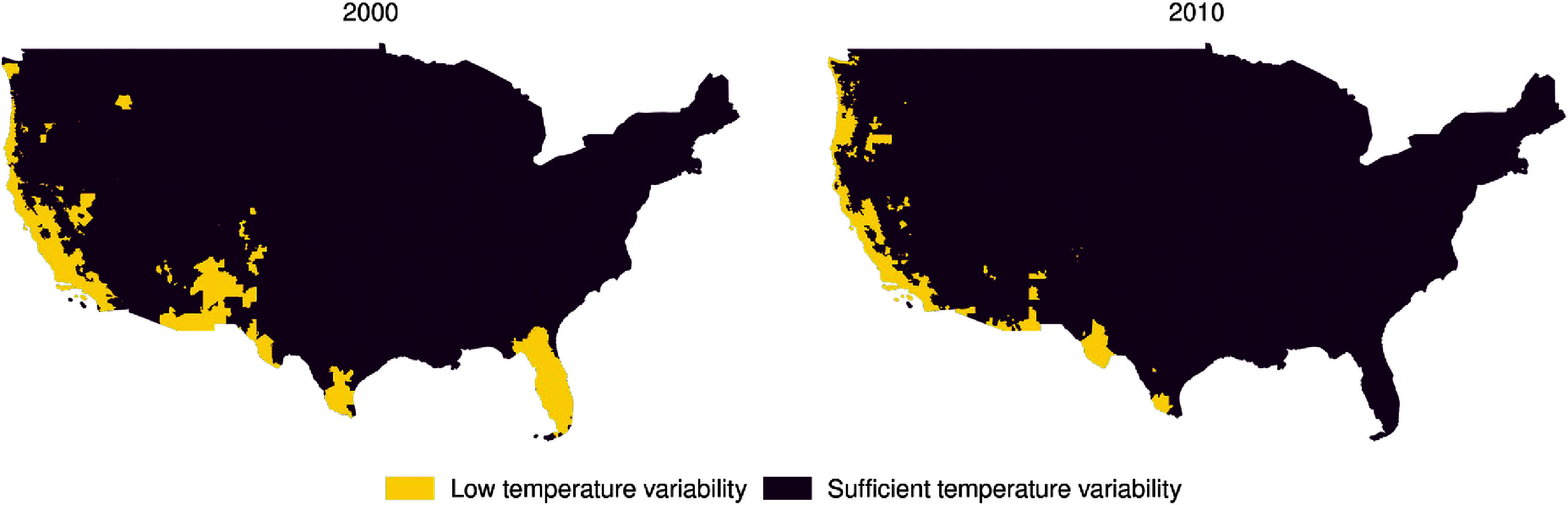
Maps showing areas of low temperature variability in the years 2000 and 2010.

In our main analysis, we excluded areas of the country with low temperature variability in order to prevent situations where a day might be considered as being both ‘extreme heat’ and ‘extreme cold’ simultaneously according to our definitions. We defined areas of low temperature variability as those where the 80th percentile of same-day minimum temperature was lower than the 20th percentile of the 5 d moving average of maximum temperature, i.e. where our loosest definitions of extreme temperature would not overlap, with some extra buffer space (figure 2). In an additional sensitivity analysis, we included all deaths from these areas.

R version 4.1.1 was used to conduct our statistical analyses (R Core Team 2021).

## 3. Results

**Figure 3.**
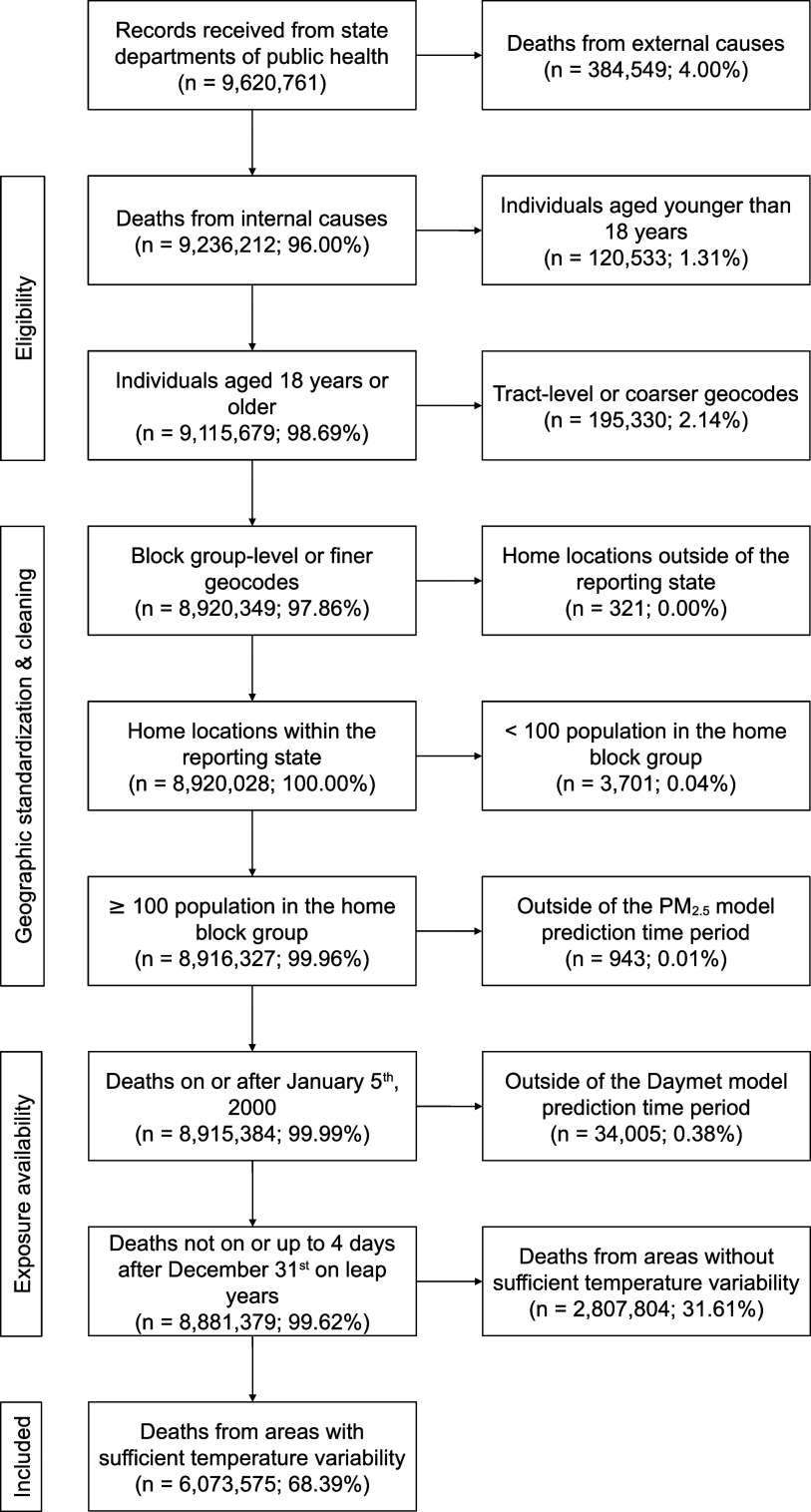
Cohort diagram showing inclusion criteria and the number of deaths dropped and retained at each step.

We obtained 9 620 761 deaths from 2000 to 2016 among the 13 states for which we had received data. From these records, we dropped, sequentially: 384 549 deaths from external causes; 120 533 deaths involving individuals younger than 18 years old; 195 330 deaths with only tract-level or coarser geocodes; 321 deaths involving individuals who resided outside of the states that reported their deaths; 3,701 deaths from block groups with populations of less than 100 each; 943 deaths that did not occur on or after January 5th, 2000, for which PM_2.5_ predictions up to the 4th lag day were not available; 34 005 deaths that occurred on December 31st of leap years, for which the Daymet model did not make predictions, up to January 4th of the next year, where predictions up to the 4th lag day were not available; and 2 807 804 deaths from areas without sufficient temperature variability. After exclusions, 6 073 575 deaths remained (figure 3).

Characteristics of included individuals at the time of death are shown in table 2. The mean age at the time of death was 75.75 years (SD: 14.66 years). Most cases involved White individuals (86.46%) and a high school degree was the most frequent highest level of educational attainment (42.64%). Most death records came from the departments of public health of Texas (24.32%), Illinois (13.97%), Massachusetts (12.81%), and Ohio (11.18%), with 37.72% of records coming from the remaining states. Eligible deaths from California and the southern border were limited by our requirement of sufficient temperature variability, which was not found in many of the heavily-populated parts of the state (figure 2 and supplement 1). Most deaths occurred in metropolitan areas (81.39%) and in cold climate zones (53.14%). The second most common Köppen–Geiger zone for deaths to occur in was temperate zones (40.74%).

**Table 2.** Characteristics of included mortality cases at the time of death, showing mean (SD) or N (%).

Characteristic	Among cases (*n* = 6,073,575)
Age (years)	75.75 (14.66)
Sex	
Male	4 918 239 (48.05%)
Female	3 155 155 (51.98%)
Unknown	181 (0.00%)
Race	
White	5 251 397 (86.46%)
Black	673 796 (11.09%)
Other	120 067 (1.98%)
Unknown	28 315 (0.47%)
Education level	
Less than high school	1 374 879 (22.64%)
High school	2 589 988 (42.64%)
More than high school	1 788 511 (29.45%)
Unknown	320 197 (5.27%)
Reporting state	
California (2009–2016)	161 901 (2.67%)
Florida (2007–2016)	454 978 (7.49%)
Georgia (2007–2009)	178 560 (2.94%)
Illinois (2008–2016)	848 656 (13.97%)
Indiana (2007–2008)	97 021 (1.60%)
Kansas (2007–2009)	61 862 (1.02%)
Massachusetts (2000–2015)	778 029 (12.81%)
Michigan (2007–2013)	546 487 (9.00%)
Missouri (2010–2016)	354 949 (5.84%)
New Hampshire (2007–2016)	85 520 (1.41%)
New Jersey (2004–2009)	349 359 (5.75%)
Ohio (2007–2013)	679 066 (11.18%)
Texas (2007–2016)	1 477 187 (24.32%)
RUCA code group	
Metropolitan (primary RUCA codes 1–3)	4 943 071 (81.39%)
Micropolitan (primary RUCA codes 4–6)	591 124 (9.73%)
Small town (primary RUCA codes 7–9)	202 169 (3.33%)
Rural (primary RUCA code 10)	337 211 (5.55%)
Köppen–Geiger classification	
A (Tropical)	60 487 (1.00%)
B (Arid)	311 133 (5.12%)
C (Temperate)	2 474 160 (40.74%)
D (Cold)	3 227 795 (53.14%)
Type of death	
Circulatory (I00-I99)	2 094 444 (34.48%)
Respiratory (J00-J99)	646 818 (10.65%)
Other internal (A00-H95 or K00-R99)	3 332 313 (54.87%)

Assessed environmental exposures for cases and controls are shown in table 3. The mean temperature was higher for controls (13.71 °C; SD: 10.64 °C) than for cases (13.64 °C; SD: 10.66 °C) and the mean ambient PM_2.5_ concentration was higher for cases (10.00 *µ*g m^−3^; SD 5.67 *µ*g m^−3^) than for controls (9.96 *µ*g m^−3^; SD: 5.65 *µ*g m^−3^), though neither of these differences were significant. The mean and standard deviation of vapor pressure were similar between cases and controls. The distributions of extreme heat and extreme cold events were also similar between cases and controls.

**Table 3.** Assessed exposures for cases and controls, showing mean (SD) or N (%). In this table, cases refer to all deaths from internal causes. Extreme heat refers to the minimum temperature on a given day being greater than the 99th percentile of minimum temperature in the same year and block group, while extreme cold refers to the maximum temperature being below the 1st percentile.

Exposure	Among cases (*n* = 6,073,535)	Among controls (*n* = 20,636,682)
Controls per case	N/A	3.38 (0.49)
Temperature [°C], same-day	13.64 (10.66)	13.71 (10.64)
2-day moving average	13.63 (10.57)	13.71 (10.55)
5-day moving average	13.60 (10.36)	13.71 (10.33)
Ambient PM_2.5_ [*µ*g m^−3^], same-day	10.00 (5.67)	9.96 (5.65)
2-day moving average	10.00 (5.18)	9.965 (5.16)
5-day moving average	9.98 (4.30)	9.96 (4.29)
Vapor pressure [kPa], same-day	1.25 (0.79)	1.26 (0.80)
2-day moving average	1.25 (0.79)	1.26 (0.79)
5-day moving average	1.25 (0.77)	1.26 (0.77)
Extreme temperature event	137 150 (2.26%)	458 912 (2.22%)
Extreme heat	63 110 (46.02%)	213 763 (46.58)
Extreme cold	74 040 (53.98%)	245 149 (53.42%)

**Figure 4.**
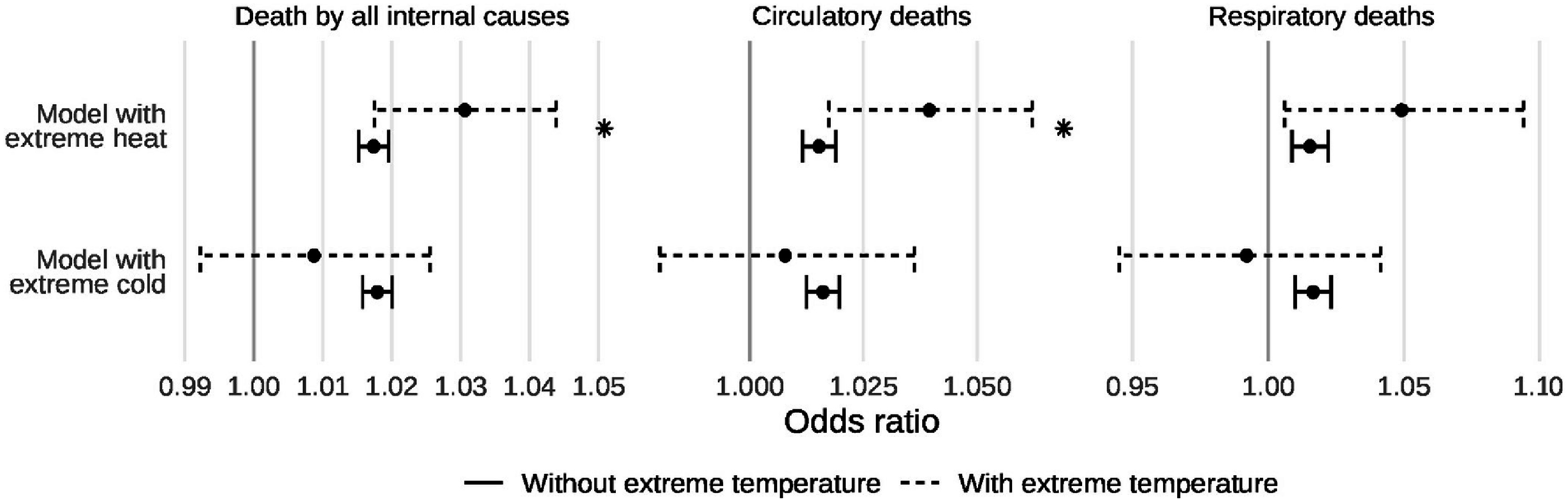
Point estimates and 95% confidence intervals of the effect of a 10 *µ*g m^−3^ increase in the 2 d moving average of PM_2.5_ on three mortality endpoints, with and without the presence of extreme temperatures. The asterisk indicates the presence of a significant interaction.

**Figure 5.**
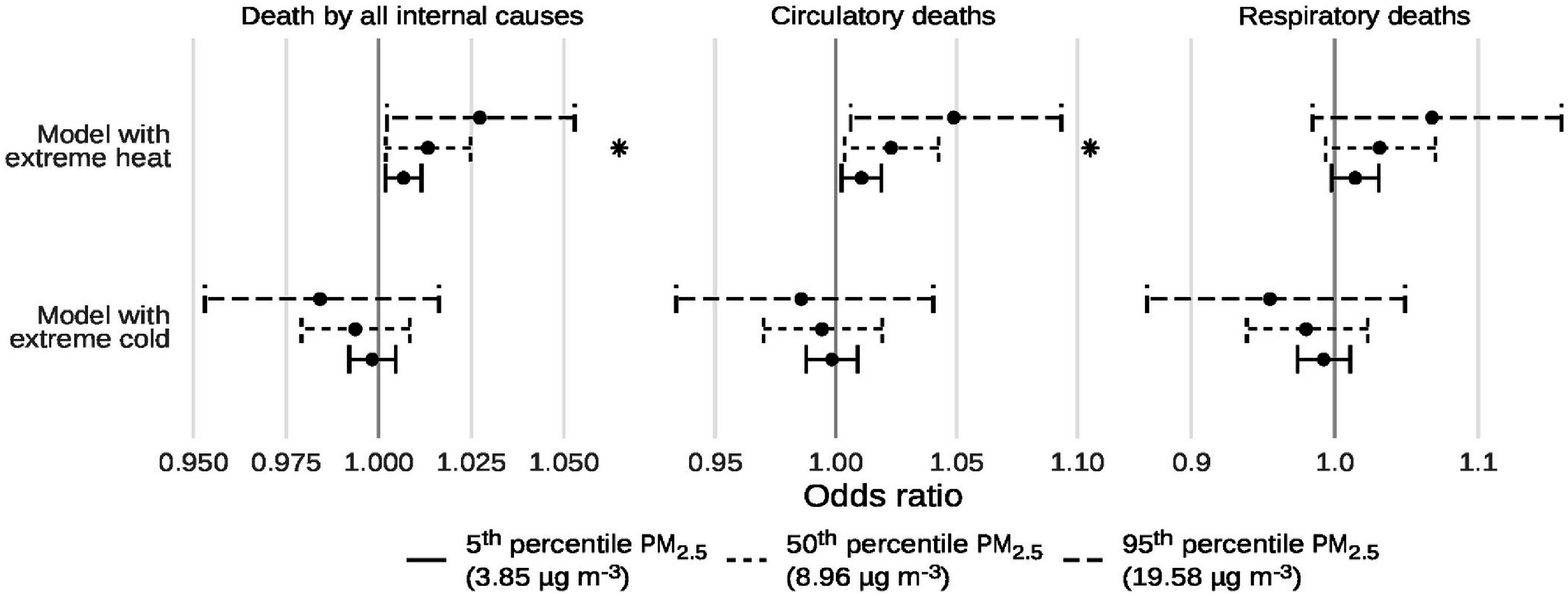
Point estimates and 95% confidence intervals of the effect of extreme temperatures on three mortality endpoints, at three different percentiles of the 2 d moving average of PM_2.5_. The asterisk indicates the presence of a significant interaction.

Results of our main analyses are shown in figures 4 and 5 and in table 4. In general, we mostly observed positive interactions between the 2 d moving average of PM_2.5_ and extreme heat and non-significantly negative interactions with extreme cold. Specifically, we observed interaction odds ratios of 1.013 (95% CI: 1.000, 1.026), 1.024 (95% CI: 1.002, 1.046), and 1.0334 (95% CI: 0.991, 1.077) for the interaction between a 10 *µ*g m^−3^ increase in the 2 d moving average of PM_2.5_ and the presence of extreme heat on deaths by all internal causes, circulatory causes, and respiratory causes, respectively. These represent 75%, 156%, and 214% increases in the coefficient estimates (log-odds ratio) for PM_2.5_ on those days compared to non-extreme heat days. The corresponding estimates for extreme cold were 0.991 (95% CI: 0.975, 1.007), 0.992 (95% CI: 0.965, 1.020), and 0.976 (95% CI: 0.930, 1.024), or 51%, 51%, and 148% reductions in the PM_2.5_ coefficient estimates (log-odds ratio) on those days. For the 2 d moving average, we only observed significant interactions with extreme heat for circulatory deaths; no significant interactions were observed for other endpoints or for extreme cold. On the additive scale, we observed significant interactions with extreme heat for deaths by all internal and circulatory causes, with RERIs of 0.013 (95% CI: 0.003, 0.021) and 0.020 (95% CI: 0.008, 0.031), respectively. Despite lack of statistical significance for all other RERIs, point estimates on the additive scale were consistently positive for extreme heat and negative for extreme cold.

**Table 4.** Estimated odds ratios and 95% confidence intervals of three mortality endpoints due to a 10 *µ*g m^−3^ increase in the 2 d moving average of PM_2.5_, the presence of extreme temperature, and the interaction between the two, and the corresponding RERIs for those interactions. Asterisks indicate *p* < 0.05.

Temperature extreme	Endpoint	Effect of PM_2.5_	Effect of temperature extreme	Interaction between exposures	RERI
Extreme heat	Death by all internal causes	1.017* (1.015, 1.020)	0.994 (0.973, 1.015)	1.013* (1.000, 1.026)	0.013* (0.003, 0.021)
	Circulatory deaths	1.015* (1.012, 1.019)	0.984 (0.949, 1.020)	1.024* (1.002, 1.046)	0.020* (0.008, 0.031)
	Respiratory deaths	1.015* (1.009, 1.022)	0.966 (0.901, 1.034)	1.033 (0.991, 1.077)	0.017 (−0.015, 0.036)

Extreme cold	Death by all internal causes	1.018* (1.016, 1.020)	1.018 (0.997, 1.039)	0.991 (0.975, 1.007)	−0.008 (−0.038, 0.011)
	Circulatory deaths	1.016* (1.012, 1.020)	1.020 (0.985, 1.056)	0.992 (0.965, 1.020)	−0.009 (−0.067, 0.023)
	Respiratory deaths	1.017* (1.010, 1.023)	1.022 (0.963, 1.085)	0.976 (0.930, 1.024)	−0.032 (−0.188, 0.018)

**Figure 6.**
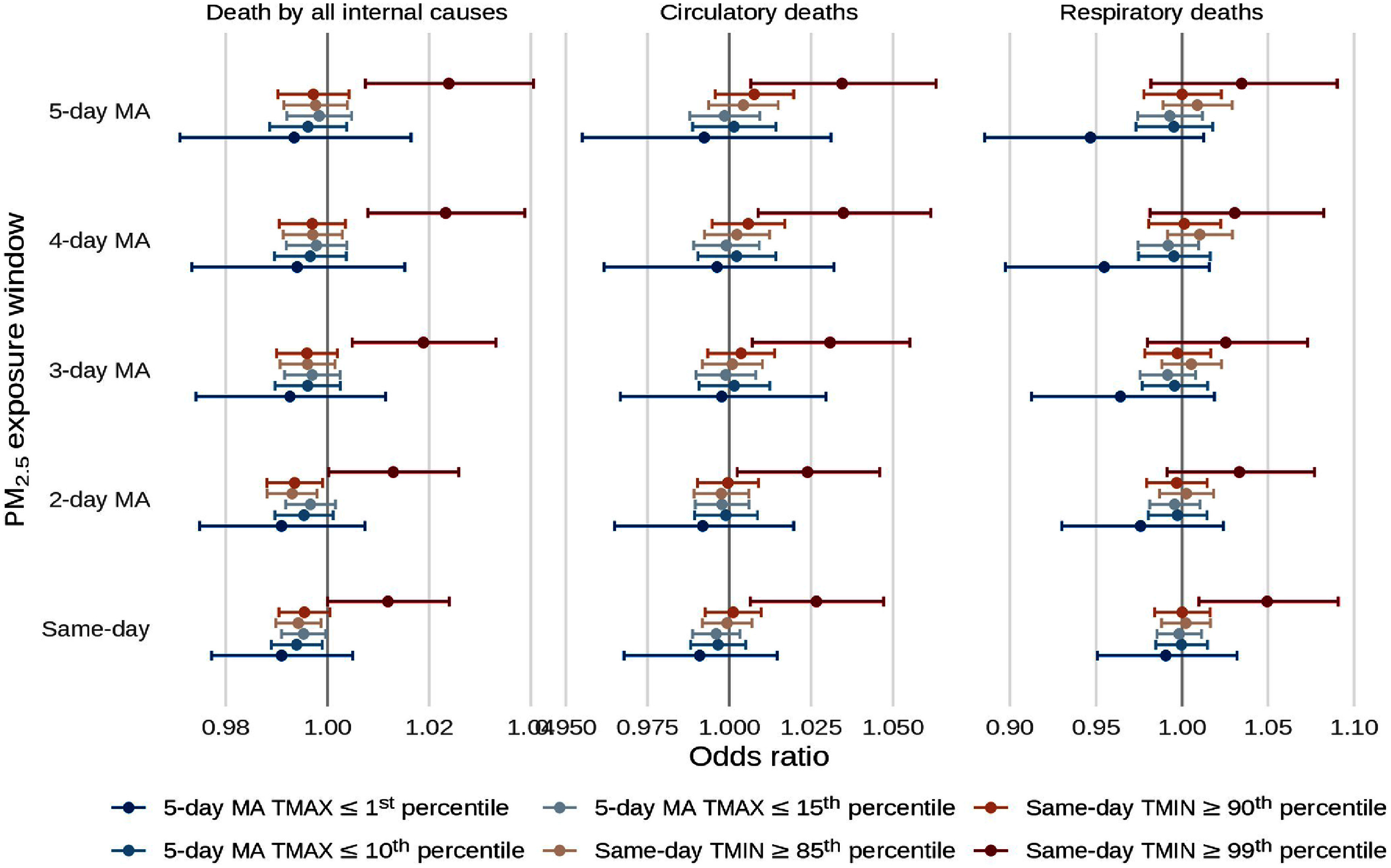
Point estimates and 95% confidence intervals for the interaction odds ratios between various moving averages of PM_2.5_ and various definitions of extreme temperature. TMIN refers to the minimum temperature, TMAX refers to the maximum temperature, and MA refers to the moving average.

Results from our sensitivity analyses looking at different definitions of extreme temperature and different moving averages of PM_2.5_ are shown in figure 6 and in supplements 2 and 3. For the strictest definition of extreme heat, we observed the strongest interactions with the 5 d moving average of PM_2.5_ for deaths by all internal causes (1.024; 95% CI: 1.007, 1.041) and the 4 d moving average of PM_2.5_ for deaths by circulatory causes (1.035; 95% CI: 1.009, 1.062). For respiratory causes, the interaction was with same-day PM_2.5_ (1.050; 95% CI: 1.010, 1.091). For the strictest definition of extreme cold, the strongest interactions were with same-day PM_2.5_ for deaths by all internal causes (0.991; 95%: CI 0.977, 1.005) and deaths by circulatory causes (0.991, 95% CI: 0.968, 1.015) while the strongest interactions were with the 5 d moving average of PM_2.5_ for deaths by circulatory causes (0.947; 95% CI: 0.885, 1.013), though these were not statistically significant. The corresponding RERIs for the aforementioned interactions between PM_2.5_ and extreme heat were also synergistic and significant (0.023; 95% CI: 0.011, 0.031 for all internal causes, 0.028; 95% CI: 0.014, 0.039 for circulatory causes, and 0.025; 95% CI: 0.007, 0.045 for respiratory causes, supplement 4). For less strict definitions of both extreme heat and cold, we observed antagonistic interactions with PM_2.5_ for deaths by all internal causes, with significant interactions between extreme temperatures and same-day or the 2 d moving average of PM_2.5_. For circulatory and respiratory deaths, the interactions between PM_2.5_ and less strict definitions of extreme heat and cold did not follow a consistent pattern.

Results from our stratified analyses are shown in supplements 5 through 10. We did not observe significant modification of either of the main effects of PM_2.5_ or extreme temperature or the interaction between the two by any grouping variable for any of the three mortality endpoints.

Results from our sensitivity analyses including deaths from areas with low temperature variability are shown in supplements 11 through 13. When including these deaths, we found that interactions between PM_2.5_ and the strictest definition of extreme heat became less synergistic while the interactions with less strict definitions of extreme heat became more synergistic. For extreme cold, we found that interactions between PM_2.5_ and all definitions of extreme cold became more synergistic for deaths by all internal causes and circulatory causes, but results were mixed for deaths by respiratory causes. All coefficients were similar to those from the main analysis.

## 4. Discussion

In this analysis, we used a large and all-encompassing collection of spatially resolved mortality records comprising over 6 million death records from 13 states across a time period of 16 yrs ranging from large cities to rural areas to evaluate the interactions between co-exposures to ambient PM_2.5_ and extreme temperatures. The size and scope of this data set has afforded great statistical power, aiding in the detection of interactions, and also aids in generalizability by capturing the entire study population over the states and years studied. In particular, we capture all residents of the states including those in smaller cities and rural areas, in contrast to many previous time series studies that only examined larger cities.

We found that recent PM_2.5_ exposure significantly increases the risk of mortality by internal causes, in line with previous studies (Di *et al* 2017). In particular, we found that the highest risk was observed for the 2 day moving average (lag days 0–1), which agrees with past literature including a large, 652-city study across 24 countries and regions that also found that the 2 d moving average had both the largest effect estimate and best fit among the different lags and moving averages used for PM_2.5_ (Liu *et al* 2019). Compared to that study, our estimates capture within-city variations in PM_2.5_ and temperature and individual events while their estimates used city-level aggregate exposure and mortality counts. The similarity of the findings provides considerable confidence that the 2 d moving average is the correct specification.

We also found that there is a synergistic interaction between PM_2.5_ and extreme heat on both the multiplicative and additive scales. We found that, in our adjusted models and sensitivity analyses, this interaction remains consistent for different moving averages of PM_2.5_, different mortality endpoints, and different subgroups of the population, though the effect becomes attenuated at less strict definitions of extreme heat. In fact, for less strict definitions of extreme heat, these interactions became antagonistic and some interactions with same-day and the 2 d moving average of PM_2.5_ were significantly so. However, overall these interactions were generally insignificant, especially for deaths by circulatory and respiratory causes, and more work must be done to investigate the robustness and cause of this phenomenon. We also found that effects and interactions were similar in smaller cities and rural areas. Results were also similar when including deaths from areas with low temperature variability.

These findings complement past studies that have also found significant modification of the effect of PM_2.5_ on mortality by ambient heat (Kioumourtzoglou *et al* 2015, Liu *et al* 2019, Steenland *et al* 2022)—particularly extreme heat (Ji *et al* 2020, Rahman *et al* 2022, Xu *et al* 2023, Zhou *et al* 2023). The exacerbation of the effects of PM_2.5_ under extreme heat conditions is biologically plausible. Prior work suggests that the interaction between heat and air pollution may be due to increased stress on and subsequent weakening of the cardiovascular system, which air pollution is independently known to affect (Liu *et al* 2019, Zhao *et al* 2021). Heat’s impacts on the respiratory system include changes such as increased ventilation rate and vasodilation, which can aid in the intake of airborne pollutants (Grigorieva and Lukyanets 2021). Particle composition can also vary by season (Requia *et al* 2019),which could also be another potential reason for the increased risks of mortality.

For extreme cold, we found suggestions of an antagonistic interaction with PM_2.5_, which was contrary to what was expected. There were not many existing studies that investigated the interaction between PM_2.5_ and extreme cold on the PM_2.5_-mortality relationship. Studies from China found that co-exposures to extreme cold and PM_2.5_ can also exacerbate the effects of PM_2.5_ on mortality, which is contrary to what we found (Huang *et al* 2023, Xu *et al* 2023). Another study from China found largely positive interactions for extreme heat but mixed results for extreme cold depending on the exposures and outcomes investigated, which was more similar to what we found in the present study (Zhou *et al* 2023). Apart from studies focused on mortality, one study from eastern China did find synergistic effects between PM_2.5_ and night-time extreme heat on the PM_2.5_-preterm birth relationship (Zhang *et al* 2023). Similar to our study, that study also found that the interaction between PM_2.5_ and extreme heat was attenuated with increasingly lenient cutoffs for what constitutes extreme heat (the 95th, 90th, and 75th percentiles of extreme heat, in that order). However, contrary to our study, that study also found synergistic interactions with extreme cold. These differences could potentially be explained by differing concentrations and characteristics of particulates between China and the U.S. compared to the present study.

The present study has several strengths. Firstly, our records comprise the entire pool of eligible deaths across the states and years covered, allowing us an unfiltered view of mortality trends not limited to a specific subpopulation. In particular we were able to access the associations in smaller communities. Second, by using block group-level geocoded deaths and 1 km resolution exposure models, we were able to capture spatial variations within urban areas in exposures, such as those that may not be evident from studies that focus on nearest monitor or city-wide averages alone. However, the use of modeled rather than directly observed exposures may also have introduced potential measurement error which may have biased our results towards the null (Wei *et al* 2022). By use of the case-crossover design, we were able to achieve robust confounding control, automatically controlling for all unobserved fixed and slow-varying factors and also those that are cyclic on a weekly period by design. However, it is still possible that some uncontrolled-for fast-varying factors may have resulted in bias. Lastly, our study population spans all deaths over the given time periods across multiple states across the country with different climates and sets of potential effect modifiers, aiding in its generalizability.

This study also has several limitations. We focused primarily on ambient temperature and PM_2.5_ conditions of the home location. Most of the time among individuals in our study population is spent indoors (Klepeis *et al* 2001), and additionally individuals may not have been at home when they died or were transported to the hospital. However, with respect to this second limitation: since our deaths involved individuals who were elderly on average, it is likely that the exposure around the home location is appropriate. We also used 1-km exposure models and standardized our mortality records at the Census block group-level, which may be too coarse to pick up on more localized patterns of adverse temperatures and elevated PM_2.5_ levels. However, these more coarse and less proximal exposures are also more robust to confounding that would occur from more personal exposures, which would require controlling for personal factors like behaviors, vehicle access, housing characteristics, etc which we may not have access to (Weisskopf and Webster 2017). Although we would have controlled for the fixed portion of these factors by our study design, the lack of data precluded studying potential effect modification by them, which is another limitation. Lastly, our definitions of ‘extreme heat’ and ‘extreme cold’ do not account for the extent of temperature variability, which necessitated excluding areas of low temperature variability from our main analysis. However, in our sensitivity analyses not excluding these areas, we found that the point estimates and confidence intervals were very similar.

## 5. Conclusions

In this study, we have demonstrated that extreme heat interacts with PM_2.5_ synergistically to produce worse outcomes than exposure to either extreme heat or ambient PM_2.5_ alone. This complements prior studies that have found similar synergistic effects between PM_2.5_ and extreme heat. These findings suggest these co-exposures warrant further attention, especially given the warming climate and also that disadvantaged and minoritized neighborhoods tend to be exposed both to more air pollution and to more extreme temperatures, particularly heat (Benz and Burney 2021, Hsu *et al* 2021, Renteria *et al* 2022). This suggests that the systemic discrimination that gave rise to present-day patterns of adverse temperature and air pollution exposures has resulted in health burdens higher than just what were caused by these two exposures independently. However, we found slight disagreements in the literature regarding the impacts and mechanisms of these interactions and further study is needed to ascertain their exact effects and functions.

## Data Availability

The data that support the findings of this study are openly available at the following URLs/DOIs: https://doi.org/10.7927/0rvr-4538; https://doi.org/10.3334/ORNLDAAC/2129; www.ers.usda.gov/data-products/rural-urban-commuting-area-codes/; www.gloh2o.org/koppen/.
